# College and Hospital Notes

**Published:** 1898-05

**Authors:** 


### COLLEGE AND HOSPITAL NOTES.

Niagara University Medical College will hold its annual commencement on Wednesday, May 11, 1898. The alumni association will meet in the morning, at which time an address of welcome will be delivered by Dr. John A. Miller, Ph. D., on the part of the faculty. The annual address of the president of the alumni association will be delivered by Dr. F. W. Maloney, of Rochester. The afternoon and evening programs will be as follows: Some remarks about incipient orthopedic cases, M. Hartwig, M. D.; Movable kidney, Wm. G. Ring, M. D.; Pathogeny of an epileptic fit, L. Pierce Clark, M. D., Craig epileptic colony, Sonyea, N. Y.; paper by L. J. McAdam, M. D., Buffalo.

Graduating exercises, Concert Hall, at 8 o'clock. Addresses by Rev. C. C. Albertson and Dr. Eugene A. Smith. Alumni banquet at the Genesee.

Physicians and all interested are cordially invited to attend these exercises.

---

The medical department of Cornell University has been established in the city of New York, with a faculty made up principally of teachers from the New York University medical school. The establishment of this new medical school has been rendered possible by the munificence of Col. Oliver H. Payne, one of New York's multimillionaires, who has presented Cornell with gifts aggregating half a million dollars in buildings, and with a considerable standing fund besides. It is expected that the doors of the new medical college will open next fall; and, as in other departments of Cornell, women will be admitted on the same terms as men.

---

The Philadelphia Polyclinic and College for graduates in medicine will devote a special week to the teaching of internal medicine and clinical diagnosis, beginning May 9 and ending May 14, 1898. The college building is at 1818 Lombard street and the fee for the course is $15.

